# Promoting Equity, Social Justice, and Saving Lives with Life Jacket and Clothing Policies

**DOI:** 10.3390/ijerph17186440

**Published:** 2020-09-04

**Authors:** Angela Beale-Tawfeeq, Linda Quan, Elizabeth Bennett, Roy R. Fielding

**Affiliations:** 1 STEAM Education Department, College of Education, Rowan University, 201 Mullica Hill Road, James Hall 2040, Glassboro, NJ 08028, USA; 2 Harborview Injury Prevention Center, University of Washington School of Medicine, Seattle, WA 98195, USA; 3 Seattle Children’s Hospital, 4800 Sand Point Way NE, Seattle, WA 98105, USA; elizabeth.bennett@seattlechildrens.org; 4 Senior Lecturer Emeritus, University of North Carolina at Charlotte, 6701 Hollow Oak Drive, Mint Hill, NC 28227, USA

**Keywords:** drowning, diverse, racial, swimming, policy, inequities, pools, swim clothing, aquatics

## Abstract

Worldwide, diverse racial/ethnic groups have disproportionately higher drowning rates. Learning to swim and wearing life jackets decrease drowning risk. We evaluated aquatic facilities’ policies regarding use of life jackets, clothing, and diapers through a lens of social justice, equity, and inclusion to ensure they met the needs of the diverse high-risk groups they serve and changing aquatic activities and programs. Public recreational pools, beach and waterpark facilities in the US and international organizations were surveyed regarding their policies on life jacket use, clothing, and diapers between 2015 and 2016. A total of 562 facilities responded, mostly pools. Almost all facilities allowed wearing life jackets in the shallow end but less so in the deep end, and wearing of T-shirts, shorts, and clothes for modesty reasons. Policies varied most on wearing non-swim clothes. Almost universal requirement of diapers applied to infants only. Respondents’ reported themes included cost, access, safety, hygiene and equipment maintenance. Reviewed policies generally reflected facilities’ responsiveness to diverse populations’ specific needs. However, policy variations around wearing clothing and swim diapers could be costly, confusing, and impede participation in aquatic activities by vulnerable populations, specifically young children and racial and ethnic minorities. Standardization of these policies could assist aquatic facilities and their users. A best-practices-based policy is outlined.

## 1. Introduction 

Drowning is a leading cause of death worldwide affecting all ages, races and ethnicities, genders, economies, and regions, but it disproportionately impacts vulnerable populations, specifically young children and racial and ethnic minorities [1]. Evidence-based interventions that could increase water safety among high-risk populations have been identified; they include learning to swim and wearing a properly fitted life jacket [2]. Swim communities have responded with drowning prevention programs geared towards culturally, linguistically, and ethnically diverse communities (CLED) (i.e., Black and African American, Indigenous, Hispanic/Latinx [3].

However, many barriers prevent the acquisition of water competency skills and experience. These barriers involve a complex web of cultural, socioeconomic, and historical factors. In the United States, racial disparities in drowning rates persist even after controlling for socioeconomic status [4]. Lack of familiarity, lack of access, fear of drowning, and the cost of attending swimming lessons and activities are barriers to learning swimming and water safety among under-resourced and CLED communities [4,5,6,7,8,9,10,11,12,13]. Furthermore, studies have shown that disparities are often systemic in nature, based on public health approaches and public policies [12,13,14,15,16]. Thus, present policies involving physical activity, health, and education in aquatics merit evaluation. 

Recreational aquatic sites have been the setting for the turmoil of social justice, water safety, and public health in the past. With CLED communities continually lacking access and having limited to no water safety education and/or drowning prevention programming, it is essential when engaging CLED communities, with policies and safety messages, that attempts be made to ensure policies and educational experiences work for all [3,4,5,6,7,12,13,14,15,16,17].

### Addressing Water Safety during a Pandemic

In recent months, multiple critical events have further underscored the need to evaluate swim-related policies. American communities are at an intersection that cannot be ignored. “America faces three major crises right now, a viral pandemic the likes of which we have not seen since 1918, an economic collapse the likes of which we have not seen since 1932 at the onset of the Great Depression, and the ongoing expressions of the struggle of civil rights and equity, the likes of which we have not seen since 1968. These are synergistic conditions, which in tandem have enormous implications for public health. They expose the continuing problem of injustice, inequality, and structural and systemic racism in America” [16].

This year, aquatic facilities, nationally and internationally, will also have in place policies to address the current novel coronavirus (COVID-19) pandemic. In the United States, recommendations will be provided by the Centers for Disease Control (CDC) [18]. Importantly, the CDC noted the increased morbidity and mortality of COVID infection among racial and ethnic minorities, as well as those with chronic medical conditions including obesity, prediabetes/diabetes, and hypertension [19]. These are the very populations who would greatly benefit from the physical activity and water competencies that aquatic facilities can provide. The CDC’s recommendations to minimize the risk of COVID-19 transmission included limiting what can be worn as swim attire and how and whether lifejackets can be used. Policies limiting options or used to minimize contamination may counter needed policies for high-risk groups in aquatic environments. 

Aquatic facilities and venues, (i.e., beaches, pools, and waterparks) will have prepared for their summer aquatic programs. Each will have individually developed policies and procedures to (a) meet educational needs to teach participants drowning prevention skills; (b) eliminate risks among high-risk participants like infants, young children, and poor swimmers; and (c) meet the specific needs of the diverse populations they serve. 

Many facilities have policies requiring specific types of swim attire; these policies may prevent vulnerable populations, specifically young children and racial and ethnic minorities from participating in aquatic physical activities, including swim programs [1]. Additionally, aquatic facilities have varying policies regarding life jacket use in the pool or at a beach, including prohibiting them. Aquatic facilities face multiple demands in setting policies: To prevent drowning, policies must make patron surveillance and safety the paramount concern. To ensure social justice, policies and procedures should not pose barriers to the use of the facility by high-risk groups. However, some demands represent conflicting priorities, exemplified by the present COVID-19 focus on minimizing viral contamination versus maximizing aquatic experiences. Aquatic policies have not been evaluated.

Nonmedical factors, social determinants of health (the circumstances in which people are born, live, and work) play an under-evaluated role in producing health and an unevaluated role in reducing drowning deaths. Addressing this, Golden et al. proposed a framework wherein health-related policies and environments were central to a healthy community [20]. Grounded in this theory-based framework of the social-ecological model, we sought to examine policies addressing life jacket, clothing, and diaper use at aquatic facilities nationally and internationally to gain an understanding of water safety policies through the lens of social determinants of health [21]. Our objectives were to assess policies addressing life jacket, clothing, and diaper use at aquatic facilities, to determine if facilities allowed the use of these items and if they imposed restrictions that might impact participants’ use of them or the facility. Our goal was that the findings from this study might further the development of standardized and more effective policies that foster the development of more culturally relevant water safety campaigns, policy, and legislation that will support wide public involvement in aquatic venues and facilities.

## 2. Methods

### 2.1. Participants

To identify approved aquatic facilities and venues, several lead organizations were contacted and agreed to distribute the survey in the United States, and internationally: for the United States, Washington State [the Washington State Drowning Prevention Network and Washington Recreational Parks Association (WRPA)] and other organizations including the American Camping Association (ACA), the American Red Cross Authorized Providers (ARCAP) and assorted US aquatic facilities (USAF); and for international assessment, the Canadian Red Cross (CRC) and the International Lifesaving Society (ILS). These lead organizations sent the survey to the public recreational (a) beach, (b) pool, or (c) water park aquatic facilities in their organizations. The study was determined to not involve human research by the Seattle Children’s Institutional Review Board which declined to review the study. 

### 2.2. Survey Instrument

The instrument used for the study was a 49-item short-response online survey (Supplementary Table S1) developed via SurveyShare, which was designed to gather descriptive information through the use of an electronic mail survey technique. To ensure comprehension for international respondents and attain high return rates, the most common challenge of surveys, survey questions were made simple, and the questionnaire brief [21]. Since most key organizations could not send out multiple survey requests, we included evaluation of Washington State facilities for whom we could repeat mailings and obtain a representative sample. 

Exploratory data was collected using a qualitative research approach, consisting of yes/no, multiple-choice, and open-ended questions. Questions focused on the type of venues (i.e., beaches, pools, and waterparks), and the following themes: (1) facility life jacket policies, (2) diaper policies and restrictions, and (3) facility clothing requirements and policies. The survey also asked respondents for a copy of their policies and explanations and comments regarding them.

A pilot study of the survey instrument was conducted to provide critique about the questionnaire format, content, expression of important items, and question deletions/additions. Content validity was presented as an overall opinion of a group of trained judges. Based on the critiques, emergent themes were identified and the survey questionnaire was revised. 

Surveys were emailed by lead organizations to their member facilities between mid-2015 and 2016. Only the Washington Diagnostic and Prevention Network (DPN) and Washington Recreation and Park Association (WRPA) distributed the survey questionnaire to their members a second time to increase response rates.

Data analysis: Simple descriptive statistics used included frequency distributions and percentages.

## 3. Results

A total of 562 survey responses were received. The response rate for Washington State facilities was 71.6%. Response rates for outside Washington State could not be calculated due to unknown denominators (Table 1). The 24 ILS respondents represented 18 countries. Most responses (80.98%) pertained to pool venues, 14.25% to beaches, and only 4.75% to water parks. Almost half the beach facilities who responded were from organizations outside North America.

Lifejackets: Almost all facilities (98.5%) allowed life jackets to be worn in the shallow end (range 88.9–98.5%). Almost all US respondents required these be US Coast Guard (USCG) approved life jackets (Table 1). While most international facilities allowed their use in the deep end, a large proportion of American facilities limited their use to the shallow end, explaining that prohibiting their use in the deep end (>5 feet) was to prevent at-risk swimmers from getting into trouble in the deep end. Respondents’ life jacket policies are listed in Supplementary Table S2.

Clothing: Most facilities allowed wearing of T-shirts (85–100%) and shorts (range 78–100%) and almost all allowed wearing clothes for modesty reasons (range 91–100%) (Table 2). However, fewer organizations allowed wearing of non-swimming clothes, (range: 33% of ILS organizations to 83.8% of Washington State facilities). While facilities cited multiple concerns, they were most likely to prohibit the wearing of street clothes worn into the facility by clients because of clothing’s perceived effect on pumps and filters. (Supplementary Table S3)

A. Supporting policies that require swim attire only:
  ○ Maintenance: clothing can cause problems with pumps (threads, etc. coming loose).
  ○ Hygiene: “Street clothes are dirty,” affect “cleanliness of the water”
  ○ Safety: Drowning risk: Loose fitting clothing is not allowed; it can cover the face; drowning rescue: “Because wet clothing weighs a lot and it’s difficult to make a rescue if needed.”
B. Supporting policies that allow other clothing, non-swim attire:
  ○ Modesty including obesity and medical issues
  ○ Cultural reasons—embracing cultural differences and embracing diversity (religious, cultural reasons)
  ○ Lack of changing facilities
  ○ Need for sun protection
  ○ Cost/disparity—“Swimwear is expensive and we want everyone to be able to swim.”

Diapers: Almost all respondents required infants to wear diapers in their pools or facilities (range 91–100%). Respondents’ diaper policies are listed in Supplementary Table S4. Although universally recommended by respondents, their required wear applied to different subgroups, including specific ages of young children, anyone incontinent, untrained, or less than fully toilet trained. The required type of diaper varied from swim diapers only to disposables with added protection to prevent leakages such as a second diaper, rubber pants, or tight-fitting swimsuit.

More than half of respondents in all categories reported providing some type of financial aid to clients to attend their programs.

## 4. Discussion

As a baseline study exploring policies addressing life jacket, clothing, and diaper use at aquatic facilities, this survey of a wide range of the US and international aquatic organizations and facilities showed that most respondents had policies that allowed the wearing of life jackets and non-swim attire clothing in their facilities’ waters. However, policies varied with the widest variations on the wearing of life jackets in the deep end and the type of clothing worn. Reasons for policy variation also varied from safety concerns for policies prohibiting life jacket use in the deep end to facility equipment concerns from street clothing debris. While most facilities allowed wearing clothing for modesty purposes, the type of clothing allowed varied among facilities. Such variations can be confusing and affect high-risk groups’ feelings of inclusiveness, comfort, financial burden, and, ultimately, the use of aquatic venues. Comments from these public facilities and the large percentage that provided some type of financial aid for the activity showed that most aquatic facilities were aware of and were attempting to address the needs of their diverse clients. While the survey reflected expanded policies to meet increasingly diverse needs, greater standardization amongst policies about what can be “worn” or used in an aquatic facility is needed.

### 4.1. Limitations

This survey study had many limitations. A convenience sample derived from several sources, it relied on large state and national organizations to identify aquatic facilities that were its members. Respondents primarily represented pool venues; waterparks and beach sites were few. To address this potentially skewed representation, we conducted a more specific evaluation of one U.S. state, Washington. However, Washington State may not be representative of the U.S. as it has focused statewide on increasing life jacket use for boating and swimming for over 20 years and has policies allowing single-gender swim that often necessitates allowing street clothing. 

Response rates were available for only Washington State as other lead organizations were unable to tell us the number of members sent surveys. Lead organizations distributed surveys to their facilities only once, except for Washington State where the lead organizations distributed the survey a second time. While respondents generally agreed, they could have represented facilities that were more likely to allow clothing or life jackets. More likely, they may have been facilities that had policies in place.

#### 4.1.1. Life Jackets 

Although life jackets are effective at preventing 50% of boating relating drowning deaths if worn [22], increasing life jacket wear among boaters has been unsuccessful except where mandated [23,24]. However, introducing and requiring their use among young children probably explains their increased, though not mandated use among young adolescents [23]. Life jacket use for swimming and playing in the water has been successful among young children; 50% of observed young children wore them in open water [25,26]. Barriers to life jacket use include knowing how to choose and fit them properly and cultural changes, and the perception that they denote an inexperienced boater or weak swimmer [27]. Thus, public aquatic facilities provide opportunities for individuals, families, and communities to become familiar with wearing and swimming in life jackets to overcome these barriers. Policies that promote life jacket use in aquatic venues may help change the culture, acceptance, and expectation that life jackets be worn. Policies that prohibit life jackets in facilities create mixed messages that confuse and could discourage use by groups most in need of added protective layers. Use of only approved life jackets should be required by facilities’ policies as unapproved buoyancy aids may appear deceptively similar to approved life jackets, are not safe, and cannot be relied upon [28]. 

#### 4.1.2. Clothing 

Clothing policies at aquatic venues have many ramifications, the first of which is water competency. Wearing clothing while attempting to self-rescue has been identified as a critical part of the basic swim competencies. [29] Many drownings occur when the victim never intended to get into the water; unintentional falls into water involve boaters, fishermen, or children on docks or wading, while wearing clothes [30].

Swimming or performing self-rescue while wearing clothes adds challenges. Evaluating physical education university students, Moran showed that wearing lightweight clothes significantly reduced swimming speed (33%) and swim endurance (28%) [31]. Evaluating their exertion levels before and after performing a range of clothing-related water activities, the young adults tested reported significantly higher exertion rating post-activity when clothed than they had estimated for all activities, irrespective of age, sex, or self-estimated water competency [32]. Importantly, clothing did not affect their ability to float. Thus, knowing that floating and swimming in clothes is possible though difficult is important to learn for self-rescue, for knowing what to expect when falling in and for the confidence to perform this skill instead of panicking. Thus, swimming while clothed was included as one of the 15 key water competencies [33]. Wearing clothes in aquatic facilities provides important experiential learning for water safety and has been incorporated into many swim programs. This may explain their acceptance among the responding aquatic facilities.

Clothing policies especially affect diverse populations at risk for drowning. These ramifications include religious rules, cultural mores, cost, and access to water safety training. Importantly, clothing/swim attire policies can be a barrier to or improve access to aquatic venues, swimming lessons, and swim experience. For some communities, swim attire needs may be religion- or culture-based. A request for swim access by Somali mothers led King County, Washington, and other cities with large Muslim populations to develop Women only swim sessions at public pools where they could be fully covered [34]. Latina and Somali mothers reported the importance of their daughters being able to wear T-shirts or other clothing and not swimming suits for modesty reasons [3]. Lastly, those who are obese represent a group that would benefit from aquatic exercise. However, in a systematic review of studies evaluating obese adolescent behaviors and thoughts regarding physical activity, swimming was specifically identified as problematic for this population [35]. They described embarrassment both when changing in the open area of aquatic facilities’ changing areas as well as when wearing swim attire. Many described wanting to wear T-shirts, a policy that was well supported by facilities we surveyed.

Thirdly, the cost of swim attire affects low-income families whose needs for swim attire grow as their children grow. Thus, clothing policies affect diverse groups in multiple and unanticipated ways and can become a barrier to the goal of increasing water safety and water recreation for all. The responses to open-ended questions revealed that some facilities accepted wearing street clothes in the pool while others sought to protect pool equipment. These different approaches should prompt further discussions within the swimming community to standardize policies. Further studies might evaluate these policies’ effects on pool equipment and participant attendance for data-driven approaches.

#### 4.1.3. Diapers

As more programs promote aquatic experiences for infants and young children, they need to protect their facilities’ water quality. While diapers were almost universally allowed and recommended by survey respondents, studies show swim diapers and swim pants may hold in some solid feces but are not leakproof [36]. Swim diapers can only delay leakage of diarrhea-causing germs, like *Cryptosporidium*, for a few minutes; they do not keep these bacteria from contaminating the water [37]. Additionally, urine contamination should be minimized as urine binds with chlorine, becoming eye and respiratory irritants [38]. Many respondents addressed this by recommending a swim diaper be used with an additional waterproof safeguard layer. The Model Aquatic Health Code (MAHC) has detailed guidelines addressing diaper use in pools [28]. Facilities should establish consistent, standardized policies regarding diapers worn in the water.

Respondents to this survey identified common themes that should be addressed in the development of an aquatic facility’s policy. Based on these common themes that were relevant across all types of aquatic venues (i.e., beaches, pools, and water parks), states and nations, and MAHC recommendations, recommendations for policies regarding life jacket, clothing, and diaper use in aquatic facilities were developed. Table 3. The effectiveness of these policies based on best practices for safety and access across communities needs to be assessed.

Public health professionals, educators and advocates, aquatic professional stakeholders, and governing bodies such as the American Red Cross play an essential role in promoting the development of best practices and recommendations regarding life jacket, clothing, and diaper policies in aquatic venues. This survey identified themes/recommendations/best practices and the need for more comprehensive and standardized guidelines for best practice.

## 5. Conclusions

Policies in pools and at beaches can facilitate or be a barrier to use and exacerbate inequities. While this study revealed a promising trend in the current use of policies that reflect facilities’ awareness of their populations’ specific needs, facilities’ policies varied. It also identified common themes, including facility maintenance-based needs such as equipment and water standards, and client-based needs such as modesty, cost, and safety, that could be used as a framework to assist policy development and assessment. Evaluating the different policies, their underlying rationale, and priorities could drive discussion and creation of best practices and standardization of current policies and laws regarding life jacket, clothing, and diaper use in aquatic facilities. Their development for water safety and drowning prevention programming could help aquatic facilities close the swimming gap among at-risk diverse and under-resourced communities. Cultural competency is the ability to understand, communicate with, and effectively interact with people across cultures. Since no one size fits all, aquatic policymakers and stakeholders should develop a dialogue with the communities they serve to assess whether they are meeting their communities’ specific needs. Additionally, this collaborative effort could also facilitate problem-solving, and promote water safety and drowning prevention efforts. Culturally competent policies with a unified set of norms and values in aquatics that reflect a commitment to access and equity could help drive needed changes in the cultural environment for drowning prevention, such as increased wearing of life jackets. The impact of COVID-19 policies on aquatic facilities underscores the need to assess and mitigate its collateral effect on access and social justice. Importantly, the impact of policies should be measured as this was not done in this study.

When looking at policies and programs, most of the time, we are looking at health behaviors, socioeconomic factors, and physical environments. Addressing disparities in drowning prevention and water safety calls for not only social but restorative justice, removal of obstacles, and development of policies that create experiences and opportunities, including social-emotional and sociocultural growth through water safety [12]. Evaluations of healthy communities demonstrate that what most impacts our health comes from outside of healthcare [39]. To address the “neglected public health threat” among CLED and other at-risk communities, it is important to investigate water safety on the larger landscape. Moreover, this needs to come from all levels of involvement and oversight, including aquatic facilities and organizations, policymakers, health departments, aquatic professionals, and communities [15,16]. Through culturally competent policies, all communities can improve access to key health determinants: physical activity/exercise, safe recreational opportunities, and water safety education in the aquatic environment.

## Figures and Tables

**Table 1:** Survey responses by major organizations/groups surveyed.

Number of Respondents						
Total 562	International Life-Saving *N* = 24 (4%)	Canadian Red Cross *N* = 149 (26.5%)	American Red Cross *N* = 99 (17.6%)	US Aquatic Facilities *N* = 190 (33.8%)	American Camping Association *N* = 32 (5.7%)	WA. State Facilities *N* = 68 (12.1%)
What type of facility do you operate?						
Beach	13 (54.2%)	7 (4.7%)	10 (10.1%)	8 (4.2%)	14 (43.7%)	14 (20.6%)
Pool	10 (41.7%)	136 (91.3%)	83 (88.5%)	166 (87.4%)	18 (56.2%)	53 (77.9%)
Water Park	1 (4.2%)	6 (4%)	6 (6.1%)	16 (8.4%)	0	1 (1.5%)
Does your aquatic facility allow life jackets?						
Yes	20 (90.9%)	145 (97.3%)	90 (95.7%)	160 (84.2%)	24 (75%)	68 (100%)
No	2 (9.1%)	4 (2.7%)	4 (4.3%)	30 (15.8%)	8 (25%)	0
Must the life jackets be US Coast Guard approved?						
Yes	16 (72.7%)	93 (65.5%)	76 (88.4%)	124 (78.5%)	18 (75%)	47 (70.1%)
No	6 (27.3%)	49 (34.5%)	10 (11.6%)	34 (21.5%)	6 (25%)	20 (29.8%)
Does your aquatic facility allow life jackets to be used in the shallow end?						
Yes	18 (90.0%)	140 (97.2%)	87 (97.8%)	152 (96.2%)	23 (95.8%)	67 (98.5%)
No	2 (10.0%)	4 (2.8%)	2 (2.2%)	6 (3.8%)	1 (4.2%)	1 (1.5%)
Does your aquatic facility allow life jackets to be used in the deep end (> 5 feet deep)						
Yes	19 (95%)	127 (88.8%)	49 (55.1%)	89 (56.3%)	20 (83.3%)	46 (67.6%)
No	1 (5%)	16 (11.2%)	40 (44.9%)	69 (43.7%)	4 (16.7%)	22 (32.3%)
Does your facility allow other types of buoyancy devices during general recreational swim, such as inflatables, foam noodles, etc.?						
Yes	19 (82.6%)	129 (87.8%)	58 (62.4%)	110 (58.2%)	24 (75%)	59 (86.8%)
No	4 (17.4%)	18 (12.2%)	35 (37.6%)	79 (41.8%)	8 (25%)	9 (13.2%)
Does your facility require infants to wear diapers in the pool?						
Yes	16 (64.0%)	135 (90.6%)	87 (93.5%)	171 (90%)	16 (50 %)	63 (92.6%)
No	9 (36.0%)	14 (9.4%)	6 (6.5%)	19 (10%)	16 (50%)	5 (7.3%)
If you answered yes to the question regarding the type of diapers is there a policy on what type of diapers?						
Yes	9 (60.0%)	94 (71.8%)	59 (72.8%)	143 (85.1%)	9 (60%)	48 (82.8%)
No	6 (40.0%)	37 (28.2%)	22 (27.2%)	25 (14.9%)	6 (40%)	10 (17.2%)
Does your facility allow clothing other than swim wear in the pool?						
Yes	11 (50.0%)	120 (80.5%)	55 (59.1%)	114 (60%)	23 (71.9%)	57 (83.8%)
No	11 (50.0%)	29 (19.5)	38 (40.9%)	76 (40%)	9 (28.1%)	11 (16.2%)
Shorts are allowed?						
Yes	9 (81.8%)	112 (94.9%)	39 (70.9%)	87 (77.7%)	22 (95.6%)	56 (98.2%)
No	2 (18.2%)	6 (5.1%)	16 (29.1%)	25 (22.3%)	1 (4.3%)	1 (1.7%)
T-shirts are allowed?						
Yes	10 (90.9%)	114 (98.3%)	52 (96.3%)	101 (91%)	23 (100%)	57 (100%)
No	1 (9.1%)	2 (1.7%)	2 (3.7%)	10 (9%)	0	0
Clothing for modesty purposes that cover the entire body is allowed?						
Yes	10 (90.9%)	110 (94.8%)	52 (94.5%)	103 (91.1%)	22(95.7%)	55 (96.5%)
No	1 (9.1%)	6 (5.2%)	3 (5.5%)	10 (8.8%)	1(43.3%)	2 (3.5%)
Do you require that the clothing be different than what the client was wearing when they came in?						
Yes	10 (76.9%)	101 (85.6%)	29 (53.7%)	53 (47.3%)	9 (39.1%)	18 (32.1%)
No	3 (23.1%)	17 (14.4%)	25 (46.3%)	59 (52.7%)	14 (60.9%)	38 (67.9%)
Does your facility have scholarships or other allowances for those who are unable to pay for swim lessons and other programming?						
Yes	14 (58.3%)	93 (62.4%)	54 (58.1%)	94 (49.5%)	23(71.9%)	52 (76.5%)
No	10 (41.7%)	56 (37.6%)	39 (41.9%)	96 (50.5%)	9 (28.1%)	16 (23.5%)

Numbers may not add up to the total number of respondents because non-respondents are not shown.

**Table 2:** Clothing for modesty purposes that covers the entire body is allowed? Responses by facility types of lead organizations/groups.

Participants	International Life Saving *N* = 11	Canadian Red Cross *N* = 116	American Red Cross *N* = 57	U.S. Aquatic Facilities N = 113	American Camping Association *N* = 23	WA State *N* = 57	Total *N* = 377	%
Pools:								
Yes	3	101	43	86	13	40	286	93.5
No	1	5	3	8	1	2	20	6.5
Water Parks:								
Yes	1	5	4	6	0	1	17	94.4
No	0	0	0	1	0	0	1	5.6
Beaches:								
Yes	6	4	7	11	9	14	51	96.2
No	0	1	0	1	0	0	2	3.8
Pools:								
Yes	3	101	43	86	13	40	286	93.5
No	1	5	3	8	1	2	20	6.5
Water Parks:								
Yes	1	5	4	6	0	1	17	94.4
No	0	0	0	1	0	0	1	5.6
Beaches:								
Yes	6	4	7	11	9	14	51	96.2
No	0	1	0	1	0	0	2	3.8

Open-ended responses fell into the following themes.

**Table 3:** Recommended guidelines for use of life jackets, clothing, and diapers in aquatic facilities.

**Life Jacket Wear in Aquatic**
● Require everyone pass a swim competency swim test to demonstrate water proficiency/competency for access to the deep end (i.e., > 5 feet) (per the American Red Cross (or similar) Swimming Competency Test) ● Require close, constant, and near supervision for those wearing approved life jackets (i.e., parents or guardians of someone wearing a US Coast Guard-approved life jacket must remain within an arm’s length of the person) ● Allow wearing U.S. Coast Guard-approved life jackets in shallow water (i.e., 5 feet or shallower or as demarcated) ● Require information on how to fit a life jacket appropriately (i.e., size ranges inclusive of infants, children, youth, individuals with disabilities, and adults) be part of making life jackets available. ● Prohibit non-USCG approved life jacket use in U.S. aquatic facilities (or similar in countries outside the U.S.)
**Use of Clothing in Aquatic Facilities**
● Require all patrons to shower for one minute, using soap, before entry. ● All swim attire or clothing worn must be free of debris, rips, and tears. ● Allow clothing worn for modesty/religious/cultural/medical purposes (i.e., Islamic, Hindu, Jewish, full cover swimwear, hijab swimwear, burkini swimwear) in shallow areas (i.e., 5 ft or less) wearing appropriate swim attire and in deep water upon successful completion of swim competency assessment.
**Use of Diapers in Aquatic Facilities**
● Require anyone who is not toilet-trained or is bowel incontinent to wear appropriate swimming diapers in or around the water. ● Promote the use of swim diapers and tight-fitting waterproof pants designed for use in and around aquatic facilities such as pools. Promote additional layering of swim diapers with waterproof diaper “covers” to minimize fecal contamination. ● Post at diaper-changing stations information or text complying with the intent of the following information: ○ Check your child’s swim diapers/vinyl pants frequently ○ Dispose of used disposable diapers in the diaper bucket or receptacle provided ○ Dispose of contents from reusable diapers into toilets and bag diapers to take home ○ Use available cleaning materials provided at the facility to sanitize the surface of the diaper-changing station before and after each use ○ Wash your hands and your child’s hands after diapering for 20 or more seconds ○ Do not swim if suffering from diarrhea

## Supplementary Materials

The following are available online at https://www.mdpi.com/1660-4601/17/18/6440/s1, Table S1: title, Video S1: List of Survey Questions; Table S2: Life Jacket policies: responses from the survey respondents; Table S3: Reported Clothing Policies; Table S4: Reported Diaper Policies.
