# Supplementary material for: Promoting Equity, Social Justice, and Saving Lives with Life Jacket and Clothing Policies
Source: Int J Environ Res Public Health. 2020 Sep 4;17(18):6440. doi: 10.3390/ijerph17186440 (PMC7559188; doi:10.3390/ijerph17186440)
Supplement: Supplementary file 1 [file ijerph-17-06440-s001.pdf]

Supplemental Table 1 – List of Survey Questions

**1) What type of facility do you operate? (Beach, Pool or Water Park)**

**2) Does your aquatic facility allow lifejackets?**

**3) Must the lifejackets be US Coast Guard approved?**

**4) Does your aquatic facility allow lifejackets to be used in the shallow end?**

**5) Does your aquatic facility allow lifejackets to be used in the deep end (greater than 5 feet of depth)?**

**6) Please add a copy of your lifejacket policy here and explain the reasoning behind your facility's policy.**

**7) Does your facility allow other types of buoyancy devices during general recreational swim, such as inflatables, foam noodles, etc.?**

**8) Does your facility require infants to wear diapers in the pool?**

**9) If you answered yes to the question regarding the type of diapers, is there a policy on what type of diapers?**

**10) What is the diaper policy and restrictions, if any?**

**11) Does your facility allow clothing other than swimwear in the pool?**

**12) Shorts are allowed?**

**13) T-shirts are allowed?**

**14) Clothing for modesty purposes that cover the entire body is allowed?**

**15) Do you require that the clothing be different than what the client was wearing when they came in?**

**16) Please explain the reason behind your facility's clothing policy**

**17) Does your facility have scholarships or other allowances for those who are unable to pay for swim lessons and other programming?**

**Supplemental Table 2: Life Jacket policies: responses from the survey respondents**

**Who can wear a life jacket:**

- Children under 13 who cannot pass a swim test must wear a USCG-approved lifejacket or be within arm's reach of a parent/guardian at all times.
- Children 6-years and younger, or children who stand less than 43 inches from the chin, must stay within the arm’s reach of a parent/guardian while in a lifejacket; Children 7-years and older, or taller than 43 inches from the chin, may swim further than an arm’s reach from a parent/guardian, but remain in the shallow-end of the pool.
- Children under 8-years old who have not passed the swim test must be within arms distance of parents or within communication distance and wearing a USCG approved life jacket
- At the lifeguards discretion they can check that the non-swimmer or swimmer needing assistance is wearing a US Coast Guard approved lifejacket.
- Children under the age of 5, a non-swimmer, or child in a lifejacket who are not proficient swimmers must be accompanied by a supervising adult within arm’s reach in the pool
- Non-swimmers shorter than 4 ft. 6 in. must wear a coast guard approved flotation device, and must be supervised by a parent in the water at a ratio of one adult to every two children
- During summer camp swim time when the ratio of child to adult is larger, we require all non-swimmers, regardless of height, to wear lifejackets until such time as they are able to pass a swimming test.
- All children under the age of 10 years who do not have a valid Morale, Welfare and Recreation (MWR) Swim License must wear a USCG approved lifejacket or be within arm’s reach of the supervising swimmer (16 years of age or older).
- For our facility, non-swimmers or weak swimmers must have in water supervision or be in a lifejacket.
- Children under 48" tall and those under 6 years of age must be accompanied by an adult in the water.
- Children under 14 who come for recreational swimming, must take a swim test either in deep water or shallow water, if they do not want to wear a lifejacket. If a child is in the water with their parent and actively being supervised, then they do not have to wear a lifejacket. If a child under 14 is in the water without their parent, but they need a lifejacket, they must take the lifejacket swim test to show that they can move through the water comfortably.

- Only non-swimmers are allowed to use personal flotation devices (PFD) (Coastguard Approved).
- Individually owned personal flotation devices or life vests shall not be worn unless approved by a pool manager on a case-by-case basis or if requested and needed as an accommodation under the Americans with Disability Act.
- All swimmers under 16 years of age will be swim tested.
- No written policy

**Type of Lifejacket:**

- "All flotation devices must be U.S. Coast Guard Approved. Water wings, baby boats, inner tubes and other related inflatables will not be allowed. YMCA life jackets are available for use upon request."
- "The only floatation devices approved for use in the pool are Coast Guard approved lifejackets, and water wings. All other floating aids are prohibited
- Use of rubber rafts, rubber boats, inner tubes, or other large floating objects, exceeding three feet in width are prohibited in designated swimming areas except U.S. Coast Guard approved life jackets, small children's floatation devices or toys and one-person inflatable mattresses for the purpose of buoyancy while swimming or playing in any designated swimming area are allowed.
- A properly fitting U.S. Coast Guard approved and labeled personal flotation device (PFD) designed to provide vertical support and to keep the head above the surface of the water may be worn, provided each non-swimmer is accompanied by an adult in the pool, within arm’s reach.
- Non-U.S Coast Guard approved personal flotation devices, inflatable toys, back floats, rafts, inner tubes, water wings, rings (including those built into bathing suits) and similar objects are not permitted in the pool.
- Lifejackets, USCG approved and properly sized to the individual, enhance the safety of bathers with less than strong swimming ability.
- LIFE JACKETS MUST BE U.S. COAST GUARD APPROVED. There is a stamp on the inside of the life jacket to show that it is CG Approved.
- Diversity Statement: Individuals with disabilities may require specialized flotation devices while at the pool. Please work closely with the patron to accommodate their needs.
- Guests may not use any outside lifejackets or flotation devices, even if they are USCG approved devices. Reason: We know the condition and safety/performance of our own lifejackets. We routinely check to ensure there is no damage to our lifejackets.

- We purchase Type 2 lifejackets that are US Coast Guard approved. For children under 50 lbs the PFDs come with head pillows, that assist with children flipping onto their back after they jump in.
- MAHC code for Wave pools - U.S. Coast Guard-approved life jackets that are properly sized and fitted shall be provided free for use by BATHERS who request them.
- Only US coast guard approved flotation devices or non-inflatable lifejackets that attach to the body are allowed We chose these two policies for a couple of reasons. One, wearing a life jacket signifies that you are a non-swimmer so in turn you need supervision in the water. Second, for the life jackets allowed we allow non-inflatable ones that attach over the shoulders and through the legs. This allows for us to allow the SPEEDO brand that is not Coast Guard Approved.
- Lifejackets, water wings, and floatation swimsuits are allowed if the children using them are within arm's reach of an adult.
- Lifejackets are allowed in the deep end; the swimmer must take the swim test in their lifejacket.
- We allow non-coast guard approved pfd's in shallow end, but only provide coast guard approved pfd's
- During open swim times, coast guard approved lifejackets must be worn by all children (under age 16) that cannot swim across the shallow end without touching the bottom. (Per our insurance company)
- No written policy

**Where to be used:**

- All non-swimmers will wear a lifejacket and may only be in shallow water at a depth they are comfortable in.
- Persons using lifejackets and water wings are not allowed in the deep end of the pool, and must be accompanied by an adult supervisor in the water at all times.
- The adult must remain in water in which they can stand flat on the pool bottom. Patrons wearing lifejackets are not permitted in deep water or on deep water play features.
- All non-swimmers and swimmers needing assistance are to stay in the shallow end of pools.
- We don't allow them in the deep end as they are still non swimmers and if they are in a lifejacket they can't touch in the shallow end so it is essentially the deep end for them.
- Deep end lifejacket use only during party rental with large inflatable toy
- Non-swimmers wearing lifejackets were not permitted to use the slide or diving board which drops into the dive tank @ 10ft in depth.
- Life jackets allowed in shallow water only (5 feet or less). Not allowed on water slides or diving boards.
- Anyone wearing a lifejacket must stay in the shallow end. If they are not comfortable swimming on their own in deeper water then they must stay where they can stand regardless of what flotation device they are using. The only time life jackets are allowed to be worn in deeper water is during a swim class.

- Life-jackets are encouraged for all. To go in the dock or in the deep water (5 ft+), they must either pass a swim test OR have a USCG approved life-jacket AND an adult.
- Lifejackets are only allowed in the deep end when the Wibit is in (Our inflatable toy).
- We do not allow any jumping into the pool with the vest on.
- We have no formal policy regarding use or disuse of life jackets.

**Exceptions and Swim Tests:**

- All youth under the age of 18 must pass a swim test prior to entering deep water. Swim test consists of a non-struggling crawl stroke (in shallow water with arms out of the water and body no more than 30 degrees) for two widths of the lap pool. Non- swimmers must stay in (chest deep) shallow water unless they are with a parent in the water in which case the water must be no more than chest deep on the parent.
- Children who have passed the swim test must be in communication distance.
- If a child under 14 is in the water without their parent, but they need a lifejacket, they must take the lifejacket swim test to show that they can move through the water comfortably.
- If a child is under the age of 6 and cannot pass a swim test, the child is required to wear a lifejacket and be within arm's reach of a parent in the water.
- If they have passed the swim test, they can wear them in the deep end. All swimmers must past the swim test to be in the deep end
- We swim test every child before they can use the deep water.
- No person unable to swim across the pool may use the pool unattended. Any adult or child may have to demonstrate his/her swimming ability before entering deep water.
- Diversity Statement: Individuals with disabilities may require specialized flotation devices while at the pool. Please work closely with the patron to accommodate their needs.
- Test: All children who are middle school age and under must be swim-tested (8th Grade and under). Mark: All children who are middle school age and under are required to wear a neck band identifying their skill level while in the pool. PFD's can be worn by all red level swimmers and must be worn by all red level swimmers in group swim time. Protect: All children middle school age and under must remain in the designated area of the pool indicated by their colored band and accompanied by a parent/guardian where necessary. Green level swimmers must complete a deep end swim test consisting of swimming 15 feet without flotation, treading water for 30 seconds, and swimming back from where they started completely unassisted and to the satisfaction of the Lifeguard administering the test. Green level swimmers will be allowed access to the entire pool area. Green level swimmers who are under middle school age (K-5th grade) must have a parent on the pool deck at ALL times. Yellow level swimmers must complete a shallow water swim test consisting of swimming the

length of the shallow end unassisted without touching the bottom or grabbing onto the wall or lane line. Yellow level swimmers will be allowed access to the shallow end only without the need of a floatation device. Parent/guardian 18 years of age or older must stay on the pool deck at all times while yellow swimmer is in the pool. Red level swimmers must be accompanied by an adult 18 years of age or older. Parent/guardian must remain in the pool within arm's reach of red level swimmer at all times. Parent/guardian cannot be responsible for more than two red level swimmers at one time. Red level swimmers must remain in the 3 feet area of the shallow end indicated by a safety line. Personal floatation devices are available for use. All groups using the pool must be tested and placed in the proper area of the pool indicated by colored neck band. All red level swimmers in the group must wear a personal floatation device at all times regardless of height while in the pool.

- All people using the deep end must pass a swim test (distance is twice the width of the pool)
- Swim Test: the swim test consists of 2 parts. First, a person must be able to swim the length of the pool (25 yards) without touching bottom, the wall, or lane ropes. Second, the person must be able to tread water for 30 seconds in the deep end of the pool.
- Swim Test: To complete the Swim Test, a participant must: Swim first half-length of lap pool on your back, any style, turn on to front, and swim remaining half-length of the pool on front with face-in, with no goggles. Immediately upon completion of the length of pool, tread water 1 minute in deep end.
- No written policy in place.

- Children under 13 who cannot pass a swim test must wear a USCG-approved lifejacket or be within arm's reach of a parent/guardian at all times.
- Children 6-years and younger, or children who stand less than 43 inches from the chin, must stay within the arm's reach of a parent/guardian while in a lifejacket
- Children 7-years and older, or taller than 43 inches from the chin, may swim further than an arm's reach from a parent/guardian, but remain in the shallow-end of the pool.
- Children under 8-years old who have not passed the swim test must be within arm's distance of parents or within communication distance and wearing a USCG approved life jacket
- At the lifeguards discretion they can check that the non-swimmer or swimmer needing assistance is wearing a US Coast Guard approved lifejacket.

- Children under the age of 5, a non-swimmer, or child in a lifejacket who are not proficient swimmers must be accompanied by a supervising adult within arm's reach in the pool
- Non-swimmers shorter than 4 ft. 6 in. must wear a coast guard approved flotation device, and must be supervised by a parent in the water at a ratio of one adult to every two children
- During summer camp swim time when the ratio of child to adult is larger, we require all non-swimmers, regardless of height, to wear lifejackets until such time as they are able to pass a swimming test.
- All children under the age of 10 years who do not have a valid MWR Swim License must wear a USCG approved lifejacket or be within arm's reach of the supervising swimmer (16 years of age or older).
- For our facility, non or weak swimmers must have in water supervision or be in a lifejacket.
- Children under 48" tall and those under 6-years of age must be accompanied by an adult in the water.
- Children under 14 who come for recreational swimming, must take a swim test either in deep water or shallow water, if they do not want to wear a lifejacket. If a child is in the water with their parent and actively being supervised, then they do not have to wear a lifejacket. If a child under 14 is in the water without their parent, but they need a lifejacket, they must take the lifejacket swim test to show that they can move through the water comfortably.
- Only non-swimmers are allow to use PFD (Coastguard Approved).
- Individually owned personal flotation devices or life vests shall not be worn unless approved by a pool manager on a case-by-case basis or if requested and needed as an accommodation under the Americans with Disability Act.
- All swimmers under 16 years of age will be swim tested.
- No written policy

**Type of Lifejacket allowed:**

- All flotation devices must be U.S. Coast Guard Approved. Water wings, baby boats, inner tubes and other related inflatables will not be allowed. YMCA life jackets are available for use upon request.
- The only floatation devices approved for use in the pool are Coast Guard approved lifejackets and water wings. All other floating aids are prohibited

- Use of rubber rafts, rubber boats, inner tubes, or other large floating objects, exceeding three feet in width are prohibited in designated swimming areas except U.S. Coast Guard approved life jackets, small children's floatation devices or toys and one-person inflatable mattresses for the purpose of buoyancy while swimming or playing in any designated swimming area are allowed.
- A properly fitting U.S. Coast Guard approved and labeled personal flotation device (PFD) designed to provide vertical support and to keep the head above the surface of the water may be worn, provided each non-swimmer is accompanied by an adult in the pool, within arm’s reach.
- Non-U.S Coast Guard approved personal flotation devices, inflatable toys, back floats, rafts, inner tubes, water wings, rings (including those built into bathing suits) and similar objects are not permitted in the pool.
- Lifejackets, USCG approved and properly sized to the individual, enhance the safety of bathers with less than strong swimming ability.
- LIFE JACKETS MUST BE U.S. COAST GUARD APPROVED. There is a stamp on the inside of the life jacket to show that it is CG Approved.
- Diversity Statement: Individuals with disabilities may require specialized flotation devices while at the pool. Please work closely with the patron to accommodate their needs.
- Guests may not use any outside lifejackets or flotation devices, even if they are USCG approved devices. Reason: We know the condition and safety/performance of our own lifejackets. We routinely check to ensure there is no damage to our lifejackets.
- We purchase Type 2 lifejackets that are US Coast Guard approved. For children under 50 lbs the PFDs come with head pillows, that assist with children flipping onto their back after they jump in.
- MAHC code for Wave pools - U.S. Coast Guard-approved life jackets that are properly sized and fitted shall be provided free for use by BATHERS who request them.
- Only US Coast Guard approved flotation devices or non-inflatable lifejackets that attach to the body are allowed We chose these two policies for a couple of reasons. One, wearing a life jacket signifies that you are a non-swimmer so in turn you need supervision in the water. Second, for the life jackets allowed we allow non-inflatable ones that attach over the shoulders and through the legs. This allows for us to allow the SPEEDO brand that is not Coast Guard Approved.
- Lifejackets, water wings, and floatation swimsuits are allowed if the children using them are within arm’s reach of an adult.
- Lifejackets are allowed in the deep end; the swimmer must take the swim test in their lifejacket.
- We allow non-Coast Guard approved pfd's in shallow end, but only provide Coast Guard approved pfd's
- During open swim times, Coast Guard approved lifejackets must be worn by all children (under age 16) that cannot swim across the shallow end without touching the bottom. (Per our insurance company)
- No written policy

**Where life jacket can be used:**

- Persons using lifejackets and water wings are not allowed in the deep end of the pool, and must be accompanied by an adult supervisor in the water at all times.
- The adult must remain in water in which they can stand flat on the pool bottom. Patrons wearing lifejackets are not permitted in deep water or on deep water play features.
- All non-swimmers and swimmers needing assistance are to stay in the shallow end of pools.
- We don't allow them in the deep end as they are still non-swimmers and if they are in a lifejacket they can't touch in the shallow end so it is essentially the deep end for them.
- All non-swimmers will wear a lifejacket and may only be in shallow water at a depth they are comfortable in.
- Deep end lifejacket use only during party rental with large inflatable toy
- Non-swimmers wearing lifejackets were not permitted to use the slide or diving board which drops into the dive tank @ 10ft in depth.
- Life jackets allowed in shallow water only (5 feet or less). Not allowed on water slides or diving boards.
- Anyone wearing a lifejacket must stay in the shallow end. If they are not comfortable swimming on their own in deeper water then they must stay where they can stand regardless of what flotation device they are using. The only time life jackets are allowed to be worn in deeper water is during a swim class.
- Life jackets are encouraged for all. To go on the dock or in the deep water (5 ft+) they must either pass a swim test OR have a USCG approved life jacket AND an adult.
- Lifejackets are only allowed in the deep end when the Wibit is in (Our inflatable toy).
- We do not allow any jumping into the pool with the vest on.
- We have no formal policy regarding use or disuse of life jackets.

**Exceptions and Swim Tests:**

- All youth under the age of 18 must pass a swim test prior to entering deep water. Swim test consists of a non-struggling crawl stroke (in shallow water with arms out of the water and body no more than 30 degrees) for two widths of the lap pool. Non- swimmers must stay in (chest deep) shallow water unless they are with a parent in the water in which case the water must be no more than chest deep on the parent.
- Children who have passed the swim test must be in communication distance.
- If a child under 14 is in the water without their parent, but they need a lifejacket, they must take the lifejacket swim test to show that they can move through the water comfortably.

- If a child is under the age of 6 and cannot pass a swim test, the child is required to wear a lifejacket and be within arm's reach of a parent in the water.
- If they have passed the swim test, they can wear them in the deep end. All swimmers must past the swim test to be in the deep end
- We swim test every child before he or she can use the deep water.
- No person unable to swim across the pool may use the pool unattended. Any adult or child may have to demonstrate his/her swimming ability before entering deep water.
- Diversity Statement: Individuals with disabilities may require specialized flotation devices while at the pool. Please work closely with the patron to accommodate their needs.
- Test: All children who are middle school age and under must be swim tested (8th Grade and under). Mark: All children who are middle school age and under are required to wear a neck band identifying their skill level while in the pool. PFD's can be worn by all red level swimmers and must be worn by all red level swimmers in group swim time. Protect: All children middle school age and under must remain in the designated area of the pool indicated by their colored band and accompanied by a parent/guardian where necessary. Green level swimmers must complete a deep end swim test consisting of swimming 15 feet without flotation, treading water for 30 seconds, and swimming back from where they started completely unassisted and to the satisfaction of the Lifeguard administering the test. Green level swimmers will be allowed access to the entire pool area. Green level swimmers who are under middle school age (K-5th grade) must have a parent on the pool deck at ALL times. Yellow level swimmers must complete a shallow water swim test consisting of swimming the length of the shallow end unassisted without touching the bottom or grabbing onto the wall or lane line. Yellow level swimmers will be allowed access to the shallow end only without the need of a floatation device. Parent/guardian 18 years of age or older must stay on the pool deck at all times while yellow swimmer is in the pool. Red level swimmers must be accompanied by an adult 18 years of age or older. Parent/guardian must remain in the pool within arm's reach of red level swimmer at all times. Parent/guardian cannot be responsible for more than two red level swimmers at one time. Red level swimmers must remain in the 3 feet area of the shallow end indicated by a safety line. Personal floatation devices are available for use. All groups using the pool must be tested and placed in the proper area of the pool indicated by colored neck band. All red level swimmers in the group must wear a personal floatation device at all times regardless of height while in the pool.
- All people using the deep end must pass a swim test (distance is twice the width of the pool)
- Swim Test: the swim test consists of 2 parts. First, a person must be able to swim the length of the pool (25 yards) without touching bottom, the wall, or lane ropes. Second, the person must be able to tread water for 30 seconds in the deep end of the pool.
- Swim Test- To complete the Swim Test, a participant must: Swim first half-length of a lap pool on your back, any style, turn on to front, and swim remaining half-length of the pool on front with face-in, with no goggles. Immediately upon completion of the length of pool, tread water 1 minute in deep end.

- No written policy in place.

**<u>Supplemental Table 3 Reported Clothing Policies</u>**

- For 20 years we did not allow clothing in the pool. Then one family complained all the way to the Director of Parks and Recreation. He changed the policy without consulting with the aquatics staff. There is no reason other than a gutless Director of Parks and Recreation
- We want to encourage people to use our facility and place as few barriers in the way as possible.
- Appropriate bathing suits/apparel must be worn while using the pools. Untorn and pre-washed t-shirts may be worn in the pool if desired. Goggles, fins, and snorkels may be used in the pool. For safety purposes, please do not use masks that cover the nose. Showers must be taken prior to entering the pool. No shoes are allowed on the pool deck. Please remove shoes in the locker room prior to entering the pool area.
- ADA and accessibility
- All guests must shower before entering facility/clothing must be worn during showering. Clothing can be at their discretion, but they must be able to swim in it.
- All pool users must wear appropriate swim attire (swimming suits and/or trunks). 1. Swim attire must be colorfast, opaque when wet and of a lightweight material suitable for swimming such as Lycra, Spandex, or Nylon. 2. Clean, colorfast t-shirts may be worn on top of swimsuits for modesty or medical reasons. 3. Clothing such as cut-off pants, basketball/athletic shorts and underwear are not permitted as swim attire. Swim attire should not be worn for exercising immediately prior to pool use for sanitary reasons. Thong bathing suits are prohibited.
- All pool users should feel comfortable and welcome when swimming at our facility.
- Appropriate swim attire is required. No fringe cut-offs allowed. No thongs or revealing attire is allowed. Rated G Facility hence rated G swimwear is required. UV protection is strongly recommended (rash guards, etc)
- As long as the article of clothing is not cotton or a cotton blend, it is permitted in the pool. Cotton fibers are known to collect and clog the pool filters. Otherwise, as long as it is "School District Appropriate" we encourage patrons to don clothing to their comfort level.
- Attire other than commercial swimwear is not allowed for the following reasons: 1. This swimwear is not made for water and can be revealing (see through/form fitting in inappropriate areas) 2. Wearing sports bras, t-shirts, spandex, or other work out attire encourages swimming right after working out, bringing sweat and bacteria into the pool 3. Shirts can become a drowning hazard if they are pulled over ones head and cling to the face 4. The chemicals can be detrimental to the clothing

- Clothes are allowed in the pool as long as they are clean. We want as many people to be able to enjoy our facilities as possible. For some, the cost of a swimsuit may be too much. Others prefer clothes for modesty or cultural reasons.
- Clothing due to religious, ethnic requirements for wearer. NOTE: Must stay in shallow end (4 feet or less).
- Clothing not intended for swimming becomes heavy and restrictive in water, and fibers that "shed" clog drains and filters. (Exceptions are made for religious attire and those who are too large to find appropriate swimwear.)
- Different cultures have different needs. We are very diverse
- Ethnic diversity... Modesty -- though we do ask that all t-shirts are light colored to avoid color bleed, require t-shirts and shorts only be worn over a bathing suit, and we don't allow "cut-off" shorts of any kind.
- Inclusive of those who may wish to be covered/wearing clothes for belief, comfort, or economic reasons.
- It clogs our filters
- Many orthodox religions require that women be covered from the neck to the ankles. Therefore Orthodox Jews, evangelical and Muslim women can wear light cotton or synthetic long sleeve shirts and pants. Also, swimmers who have body image issues may wear appropriate t-shirts.
- Modesty for women. Cleanliness for men (bringing dirty street clothes into the water).
- Must shower completely before entering pool, regardless of attire. Have special use sometimes by military. Allow shirts and/or rashguard, not denim shorts or sweats.
- Our policies are based on what a reasonable person would agree to
- RULE: Indy Parks and Recreation Aquatic Facilities are family facilities: No inappropriate behavior, language or swimwear. No undergarments or street clothes may be used as swimwear. WHY: We strive to provide an atmosphere that is welcoming to everyone. The Marion County Health Department regulations state no undergarments or street clothes. Rash guard shirts are a new trend and are considered swimwear. These shirts should fit the swimmer and not be baggy. WHAT IS INAPPROPRIATE SWIMWEAR? See-through clothing, underwear, dirty clothing, indecent coverage ENFORCEMENT: Inappropriate behavior and language should be stopped right away. Follow the Correcting Behavior Guidelines. Cashiers should be asking patrons about proper swim wear as they enter the facility but sometimes people slip through. If you are guarding and notice inappropriate swimwear, get a supervisor. The guest may be offered a comp pass. PLEASE SEE DIVERSITY STATEMENT ABOUT SWIMWEAR EXCEPTIONS. Diversity Statement: Expect a diverse population at your facility. We welcome people of any race, color, religion, sex, national origin, age, ability, or sexual orientation. We strive to make our pools accessible to everyone. There may be exceptions to the general Indy Parks Aquatic Policies due to religious, cultural, or medical reasons. Customers who wear full coverage clothing or t-shirts for religious, cultural, medical, or modesty will be allowed in the shallow areas of pools only. They may not use any slide, climbing wall, or diving board. This is for their safety.

- They can wear clothing but has to meet the following criteria: All clothing must be colorfast and lightweight material suitable for swimwear, such as Lycra, Spandex or nylon. So you might see an overweight person wear gym clothes (yoga pants) instead of a bathing suit.
- VERY diverse population: ethnicity, economically, religiously. Clothing must be appropriate (no white t-shirts), no cut- offs, no snaps, buckles, zippers, rivets, etc. to scratch or damage the slides.
- We allow clothing that does not hinder the patrons ability to swim safely
- We are not a washing machine! We do allow swim shirts.
- With so many people, it would be difficult to enforce swim suits only
- We believe that clothing that is not designed for swimming could be a drowning hazard.

**Supplemental Table 4: Reported Diaper Policies**

Who should wear diapers:

- Children who are not toilet-trained must wear swim diapers
- Non-potty-trained swimmers are required to wear a swim diaper under their swim suits.
- All children under 3
- Anyone not fully toilet trained
- Anyone not toilet-trained or incontinent entering the pool
- Approved Swim Diapers must be worn by swimmer who still require the use of diapers.
- All children who are age 3 and under and all children over age 4 and not completely potty trained must follow our double diaper policy.
- For all children under the age of 4
- Anytime a child under the age of 2 enters the facility the cashier should ask the adult if they have a swim diaper.
- No policy in place

What kind of diaper allowed:

- Swim diapers with tight fitting rubber pants over the top.

- Wear plastic pants or a tight-fitting swimsuit
- Disposable diapers are not acceptable because they fall apart in the water.
- Swim diapers and rubber pants
- We sell swim diapers at our front counter for parents who may not have one with them.
- Swim diaper and plastic pants over the swim diaper.
- Wear a diaper approved for use in water
- Wear either an appropriately sized swim diaper or a cloth diaper with a tight-fitting plastic covering for both legs and waist
- Wear a swim diaper, either disposable or reusable.
- Must wear a clean swim diaper covered with separate waterproof pants, all of which must fit snugly around the legs and waist and under the swimsuit. Standard diapers are not permitted.
- No thongs or revealing attire is allowed. *Infants and toddlers who are not potty trained must wear plastic pants or a tight-fitting swimsuit. *Disposable diapers are not acceptable because they fall apart in the water
- Children wearing diapers must have vinyl pants over the diaper and under the bathing suit. The Club reserves the right to ask for a child to be removed from the water if they do not have on vinyl pants. Vinyl pants may be purchased from the Club for $2.
- Conform to local health code. Snug-fitting plastic type elastic diaper covering.
- Must wear swimming diapers or a cloth diaper with a plastic cover that has tight fitting legs. No regular disposable diapers or loose fitting swim suits are allowed. Disposable swim diapers for sale at cashier booth.
- Aquatic diapers
- Diapers manufactured for swimming.
- Diapers must be aquatic disposable diapers
- Double diaper. Disposable swim diaper first then over that a pair of rubber pants or non-disposable swim diaper with elastic that goes around the legs and waist.
- Must wear a swim diaper covered by a waterproof, leak-proof vinyl diaper cover or swimsuit with liner.
- Must have a swim diaper or tight-fitting plastic pant over a standard diaper.
- Swimmies and rubber pants, but no one uses the rubber pants
- We adhere to the Washington state health code. Our policy states the following:  A swim diaper with a protective plastic covering is required for anyone not potty trained Usually this requires a disposable swim diaper to be worn with a protective plastic diaper over the top.
- We don't have an exact policy on swim diapers. We do sell the "little swimmers" and honestly, we have a good profit margin on them.

- We recommend swim diapers and a plastic tight legged cover (plastic pants) as stated by our state health dept code for water recreation facilities. And we sell both at our front desk. Our front desk staff is great at educating public about this (even though the public doesn't listen most of the time).
